# Electrical properties and synaptic transmission in mouse intracardiac ganglion neurons *in situ*

**DOI:** 10.14814/phy2.15056

**Published:** 2021-09-28

**Authors:** Alexander A. Harper, David J. Adams

**Affiliations:** ^1^ Illawarra Health and Medical Research Institute (IHMRI) University of Wollongong Wollongong New South Wales Australia

**Keywords:** electrical properties, intracardiac neuron, nicotinic acetylcholine receptor, synaptic transmission, vagal stimulation

## Abstract

The intrinsic cardiac nervous system represents the final site of signal integration for neurotransmission to the myocardium to enable local control of cardiac performance. The electrophysiological characteristics and ganglionic transmission of adult mouse intrinsic cardiac ganglion (ICG) neurons were investigated using a whole‐mount ganglion preparation of the excised right atrial ganglion plexus and intracellular microelectrode recording techniques. The passive and active electrical properties of ICG neurons and synaptic transmission including synaptic response strength and efficacy as a function of stimulation frequency were examined. The resting membrane potential and input resistance of ICG neurons were −47.9 ± 4.0 mV and 197.2 ± 81.5 MΩ, respectively. All neurons had somatic action potentials with overshoots of >+15 mV and after‐hyperpolarizations having an average of 10 mV amplitude and ~45 ms half duration. Phasic discharge activities were recorded from the majority of neurons studied and several types of excitatory synaptic responses were recorded following inputs from the vagus or interganglionic nerve trunk(s). Most postganglionic neurons (>75%) received a strong, suprathreshold synaptic input and reliably followed high‐frequency repetitive nerve stimulation up to at least 50 Hz. Nerve‐evoked synaptic transmission was blocked by extracellular Cd^2+^, ω‐conotoxin CVIE, or α‐conotoxin RegIIA, a selective α3‐containing nicotinic acetylcholine receptor antagonist. Synaptic transmission and the electrical properties of murine ICG neurons contribute to the pattern of discharge which regulates chronotropic, dromotropic, and inotropic elements of cardiac function.

## Graphical abstract

The intrinsic cardiac nervous system represents the final site of signal integration for neurotransmission to the myocardium to enable local control of cardiac performance. The electrical properties and synaptic transmission of adult mouse intracardiac ganglion neurons were characterized in a whole‐mount preparation of the excised right atrial ganglion plexus.

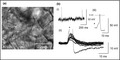

## 1 INTRODUCTION

Neural control of the vertebrate heart is under the influence of both the sympathetic and parasympathetic divisions of the autonomic nervous system. The mammalian parasympathetic projection originates in the nucleus ambiguus and dorsal motor nucleus of the brain stem. The preganglionic axons originating from these motor nuclei travel in the vagus nerve. Vagal control of the heart involves the convergence and integration of projections from the vagal motor nuclei within the intracardiac ganglia (ICG) forming the final common pathway for the cardiac autonomic nervous system (Ardell & Armour, 2016; Fedele & Brand, 2020; Wake & Brack, 2016). ICG are interconnected clusters of neurons located throughout the atrial epicardium and inter‐atrial septum (Pauza et al., 2013; Zarzoso et al., 2013). They are innervated by the vagus nerve and send their projections to discrete regions of the heart. The ICG play a pivotal role in neural control of cardiac function and the final pattern of discharge in ICG neurons regulates chronotropic, dromotropic, and inotropic elements of cardiac function (Adams & Cuevas, 2004). Mechanical disruption or pharmacological blockade of parasympathetic innervation has been reported to shorten ventricular refractory periods and increase the incidence of ventricular arrhythmia in murine hearts (Herring et al., 2019; Jungen et al., 2017). Many studies, using diverse recording techniques, species, and levels of cellular organization have considered the properties of mammalian intrinsic cardiac ganglion neurons (Ashton et al., 2018).

There have been several reports characterizing the phenotypic properties of mouse ICG neurons, however they have been largely limited to immunohistochemical and electrophysiological studies on dissociated neurons in culture (Fregoso & Hoover, 2012; Hoard et al., 2007, 2008). There is accruing evidence that the expression and distribution of membrane receptors and ion channels in dissociated autonomic ganglion neurons are not necessarily similar to that of the neurons in the intact ganglion. For example, ATP (P2X) receptor channels are not expressed in the whole‐mount postganglionic submandibular neurons (Smith et al., 2001) but are present in dissociated neurons from these ganglia (Liu & Adams, 2001). In contrast, apamin, a small conductance Ca^2+^‐activated K^+^ (SK) channel inhibitor, produced no change in firing discharge in dissociated neonatal rat ICG neurons (Cuevas et al., 1997), whereas in ICG neurons in both adult and neonatal rat ganglion preparations, apamin reduced after‐hyperpolarization (AHP) duration and switched the discharge characteristics from phasic to tonic (Rimmer & Harper, 2006).

Several animal models have been used to investigate ganglionic transmission in the ICG; in isolated preparations: rats, adult (Selyanko & Skok, 1992) and neonatal (Seabrook et al., 1990), guinea pigs (Edwards et al., 1995), and *in vivo*: dogs (Bibevski et al., 2000; Xi‐Moy et al., 1993) and pigs (Smith, 1999). A key milestone study used the working heart‐brainstem preparation (WHBP) in young rats for an intracellular analysis of the action of cervical vagal electrical stimulation and application of strong reflex stimuli (baroreceptor and peripheral chemoreceptor) upon synaptic activity and characteristics in ICG neurons (McAllen et al., 2011). The WHBP makes it possible to investigate the reflex pathways influencing cardiac vagal activity in the absence of the effects of anesthetic, for example, any ongoing vagal tone.

To the best of our knowledge, there are no studies on synaptic transmission in mouse ICG neurons and a key feature of the present investigation is the use of an intact ganglion preparation. We have investigated the intrinsic membrane properties and postsynaptic responses to vagal nerve stimulation in adult mouse ICG neurons. An isolated whole‐mount preparation was used comprising the right atrial ganglionic plexus and underlying myocardium. Consideration of the reported electrophysiological characteristics of mammalian ICG neurons reveal several significant differences. A catalog of the range of electrical and synaptic properties of mouse ICG neurons is therefore a fundamental requirement before the use of genetically modified animal models. A preliminary account of some of the results has been presented as a published abstract (Harper & Adams, 2019).

## 2 METHODS

### 2.1 Whole‐mount intracardiac ganglion preparation

The whole‐mount ICG preparation has been described previously for the rat (Rimmer & Harper, 2006). Young (8–10 week old) adult male C57BL/6NJAusb mice (Australian BioResources) were terminally anesthetized with Fluothane as approved by the University of Wollongong Animal Ethics Committee (AE16/10). The heart and lungs were quickly excised and the right atrial ganglion plexus and underlying myocardium were isolated from the dorsal surface of the atria.

A whole‐mount preparation was pinned out in a recording chamber (∼1 ml volume) lined with Sylgard 184 silicone elastomer (Dow Corning) and superfused with bicarbonate‐buffered physiological salt solution (PSS) at ∼1 ml/min by gravity. The temperature of the superfusing solution was controlled by a Peltier heating device (PDMI‐2 micro incubator; Medical Systems Corp.) to 36°C, monitored by an independent thermistor probe in the recording chamber. The tissue was left to resuscitate in these conditions for ∼30 min before commencing electrophysiological recording. ICG neurons were visualized using differential interference contrast (DIC) optics on a fixed stage microscope. Recordings were normally made from the sinoatrial ganglion, the largest located at the junction of the right superior vena cava and right atrium (Sampaio et al., 2003).

### 2.2 Electrophysiological recording, data acquisition, and analysis

Intracellular recordings from postganglionic ICG somata were made using sharp microelectrodes pulled from borosilicate glass (GC120F; Harvard Apparatus) with DC resistances of ≥120 MΩ when filled with 0.5 M KCl. This filling solution has been widely used in studies of mammalian autonomic ganglion neurons (e.g., Edwards et al., 1995). Membrane voltage responses were recorded with a single microelectrode clamp amplifier used in Bridge mode, NPI SEC‐05X (npi electronic GmbH). Voltage and current signals were digitized at 50 and 10 kHz, respectively, and transferred to a computer using an analog‐to‐digital converter [Digidata 1322A/Clampex 9.2 acquisition systems (Molecular Devices)].

Branches of the vagus and interganglionic nerve trunks were stimulated using a tight fitting suction electrode fabricated from borosilicate glass (GC150F; Harvard Apparatus) connected to a constant voltage isolated stimulator (Digitimer DS2; Digitimer Ltd.). Nerve trunks were stimulated using stimulus pulses of twice threshold voltages, 0.02–0.2 ms width and 5–90 V amplitude.

The voltage difference between the microelectrode and the bath electrode was measured at the end of each recording, having been zeroed before impalement. Recordings were discarded if this potential proved to be >±5 mV. Membrane voltage responses were recorded in conventional Bridge mode. Two types of protocol were routinely performed. Brief intracellular depolarizing currents 2 ms in duration were used to evoke single somatic action potentials. Long (500 ms) hyperpolarizing and depolarizing current (0.1 nA increment steps) were used to measure time‐ and voltage‐dependent rectification and evoked discharge, respectively.

The Bridge was balanced both before and following impalement of the neuron. A concern in single microelectrode studies is that the current pulse injected equates to that intended, especially with high‐resistance electrodes. The small fraction of records in which there was a manifest alteration in microelectrode resistance during current injection, as evidenced by poor balancing characteristics, was discarded.

The peak, with time, voltage response to long hyperpolarizing pulses (−0.1 nA) was used to measure input resistance (*R*_in_) and time constant (*τ*). The latter was measured by fitting ~20%–80% of the rising phase with a single exponential function using Origin 2018 software (Origin Lab Corp.). Membrane resistance (*R*_m_) was calculated from *R*_in_ × cell capacitance (*C*_in_, assuming 1 pF = 100 µm^2^).

### 2.3 Solutions and pharmacological agents

Physiological salt solution (PSS) contained (in mM): 118 NaCl, 25 NaHCO_3_, 1.13 NaH_2_PO_4_, 4.7 KCl, 1.8 CaCl_2_, 1.3 MgCl_2_, 11.1 d‐glucose, and was gassed with carbogen (95% O_2_–5% CO_2_) to pH 7.4. All reagents were of analytical grade. In many preparations, atrial contractions presented a challenge to intracellular recording and in these instances contractions were suppressed by blebbistatin (5 µM; Cayman Chemical Co). Blebbistatin was dissolved in dimethyl sulfoxide (DMSO) for a stock concentration of 20 mM and further diluted with PSS to 5 µM, the final concentration of DMSO was 0.025% v/v. Blebbistatin, a cell‐permeable inhibitor of myosin II ATPase, was used for its negative inotropic activity, curtailing atrial contractions to allow stable intracellular recordings. Blebbistatin, at similar concentrations to that used in this study, had no effect on the atrial and ventricular action potential of zebrafish embryonic heart (Jou et al., 2010) and no significant effect on the conformation of the action potential of human‐induced pluripotent stem cell‐derived cardiomyocytes (Hortigon‐Vinagre et al., 2021).

α‐Conotoxins RegIIA and ImI were synthesized and kindly provided by Dr R. Yu (Ocean University of China), and ω‐conotoxin CVIE was synthesized and provided by Prof P. F. Alewood (University of Queensland).

### 2.4 Data and statistical analysis

Data are presented as the means ± SD of the number of observations (individual neurons, *n*) indicated, and were compared using Student's *t*‐tests as indicated in the text (Prism 8, GraphPad Software, Inc.) using a statistical significance (*) of *p* < 0.05.

## 3 RESULTS

**FIGURE 1:**
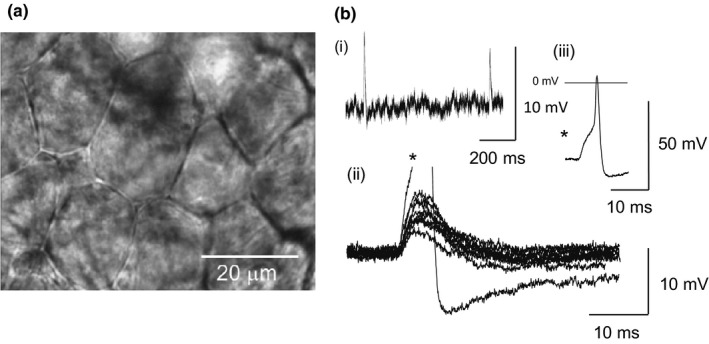
(a) Intrinsic cardiac neurons in a living adult mouse right atrial ganglion preparation viewed with differential interference contrast optics. (b) Spontaneous activity: (i) trace of spontaneous EPSPs in ICG neuron, (ii) overlaid 10 consecutive spontaneous EPSPs in the same neuron showing the spectrum of amplitudes, one of which exceeded threshold, * (iii) spontaneous action potential from * displayed on distinct scaling

All results are from ICG neurons with a resting membrane potential more negative than −40 mV and overshooting somatic action potentials evoked by 20 ms, +0.2 nA depolarizing pulses. The photomicrograph shown in Figure 1a depicts intrinsic cardiac neurons in an adult mouse sinoatrial ganglion preparation. This large ICG was located at the junction of the right superior vena cava and right atrium, and in the rat stimulation of this ganglion principally evokes a bradycardic response (Sampaio et al., 2003). The ganglionic neurons and surrounding satellite glial cells form a flat sheet on the surface of the underlying atrial muscle tissue. Some neurons of the in situ preparation exhibited spontaneous excitatory synaptic activity and, occasionally, spontaneous action potentials (APs) (see Figure 1b). Spontaneous events were present in 5/23 neurons recorded from at least six different mouse ICG preparations. In contrast, with the reports for guinea pig ICG (Edwards et al., 1995), no instances of pacemaker‐like AP discharge activity were recorded which is in agreement with a previous report on rat ICG (Rimmer & Harper, 2006).

### 3.1 Postganglionic neuron properties

**FIGURE 2:**
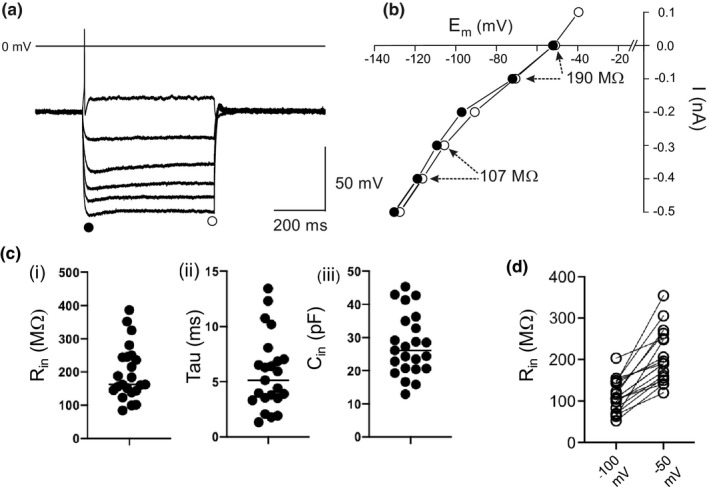
(a) Representative voltage traces in response to long 500 ms depolarizing (+0.1 nA) and hyperpolarizing (−0.1 to −0.5 nA) current pulses. (b) Corresponding current–voltage plot measured as the peak, with time, responses (●) and at the end of the current pulse (○). (c) Scatterplots of (i) input resistance (*R*_in_), (ii) time constant (Tau), and (iii) cell capacitance (*C*_in_). Line denotes mean value. (d) *R*_in_ measured at resting membrane potential ~−50 and −100 to −120 mV

The mean resting membrane potential was −47.9 ± 4.0 mV (*n* = 25) in good agreement with those reported for ICG neurons in adult rat and guinea pig intact ganglia in comparable experimental conditions (Dyavanapali et al., 2009; Edwards et al., 1995; Selyanko, 1992) and in the WHBP (McAllen et al., 2011). Representative voltage responses for long duration depolarizing (+0.1 nA) and hyperpolarizing current steps (−0.1 to −0.5 nA) for a mouse ICG neuron and corresponding current–voltage (I–V) plot are presented in Figure 2a,b. The mean input resistance (*R*_in_) was 197.2 ± 81.5 MΩ and time constant (*τ*) was 5.8 ± 3.4 ms (*n* = 23) were measured using the −0.1 nA hyperpolarizing current step, minimizing the contribution of voltage‐dependent conductances. From this cell capacitance (*C*_in_) and membrane resistance (*R*_m_) were calculated to be 27.7 ± 9.3 pF and 5.8 ± 3.4 kΩ·cm^2^ (*n* = 23), respectively. Scatterplots of *R*_in_, *τ*, and *C*_in_ are shown in Figure 2c.

Hyperpolarizing pulses can induce inward voltage‐ and/or time‐dependent rectification (TDR) in rat ICG neurons (Hogg et al., 2001). TDR was evident as a sag in the voltage response to a hyperpolarizing current step (Figure 2a). Peak and steady‐state values for the voltage response are plotted in Figure 2b with the steady‐state value being reduced to 0.88 ± 0.06 (*n* = 23) of the peak value (*p* < 0.0001, paired *t*‐test). TDR is held to be the signature of the hyperpolarization‐activated cation current (H‐current) (Biel et al., 2009; Gao et al., 2012). The degree of TDR was determined from the steady‐state voltage response to a hyperpolarizing current to approximately −100 ± 10 mV and expressing this as a decimal of the peak, with time, membrane potential excursion. This was 0.94 ± 0.01 (*n* = 3) of control values which was abrogated by superfusion of 2 mM Cs^+^ consonant with the H‐current underpinning TDR in these neurons.

Voltage‐dependent rectification (VDR) was observed in all neurons. The extent of VDR was quantified by measuring the resistance of the I–V plot at resting membrane potential (−50 and −120 mV, see Figure 2d). Slope resistance taken at these values is presented in Figure 2d. The values at resting membrane potential were significantly different from those at hyperpolarized membrane potentials, being 205.7 ± 63.5 MΩ and 113.2 ± 39.0 MΩ, respectively (*n* = 18, *p* < 0.0001, paired *t*‐test). Membrane hyperpolarization reduced *R*_in_ ~twofold, consistent with an increase in membrane conductance in mouse ICG neurons (Figure 1d).

### 3.2 Evoked discharge and active action potential properties

**FIGURE 3:**
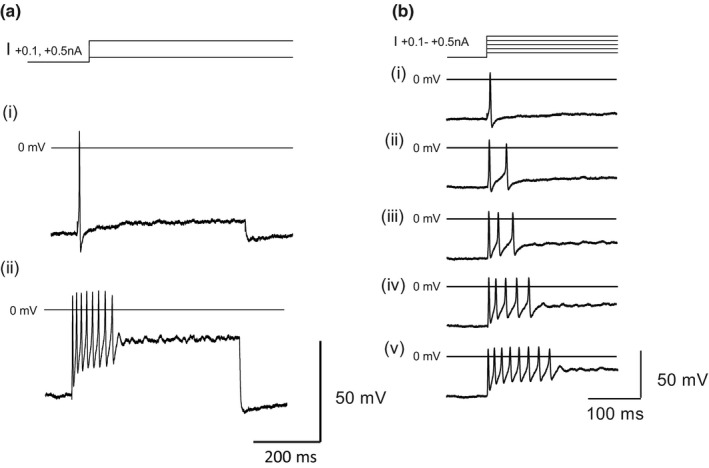
(a) Illustration of the voltage responses to long depolarizing current pulses (500 ms, +0.1, and +0.5 nA) from an adult mouse ICG neuron. (b) (i–v) Discharges evoked by depolarizing currents +0.1 to +0.5 nA shown at a faster sweep rate

To characterize firing discharge, long (500 ms) depolarizing current pulses were used and representative examples are shown in Figure 3. Adult murine ICG neurons displayed a predominantly phasic discharge. The number of action potentials fired at +0.2 nA ranged between 1 and 4 (median 1, *n* = 22).

**FIGURE 4:**
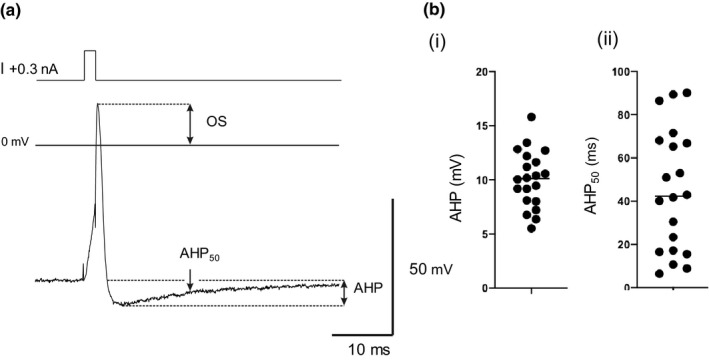
(a) Somatic action potential trace produced by injection of a brief pulse (+0.3 nA, 2 ms) from adult mouse ICG neuron. Action potential characteristics measured were: overshoot (OS), after‐hyperpolarization (AHP), and recovery of the AHP (duration to 50% recovery), AHP_50_. (b) Scatterplots displaying (i) the distribution of AHP and (ii) AHP_50_ recovery values. Line denotes mean value

A representative somatic action potential (AP) evoked by a short (2 ms, +0.3 nA) current pulse is shown in Figure 4a. The AP overshoot (OS) was 16.7 ± 5.9 mV (*n* = 20). The after‐hyperpolarization (AHP) following the AP was characterized by its amplitude and duration (time to 50% recovery, AHP_50_). Scatterplots for AHP amplitude and AHP_50_ values are shown in Figure 4b. APs in mouse ICG neurons had small amplitude AHPs, ranging from 5.5 to 15.8 mV with a mean value of 10.0 mV (*n* = 20). In contrast, the scatterplot of AHP_50_ values reveals a wide range of 6.4–90.2 ms, the mean duration being 44.8 ms.

### 3.3 Ganglionic transmission

**FIGURE 5:**
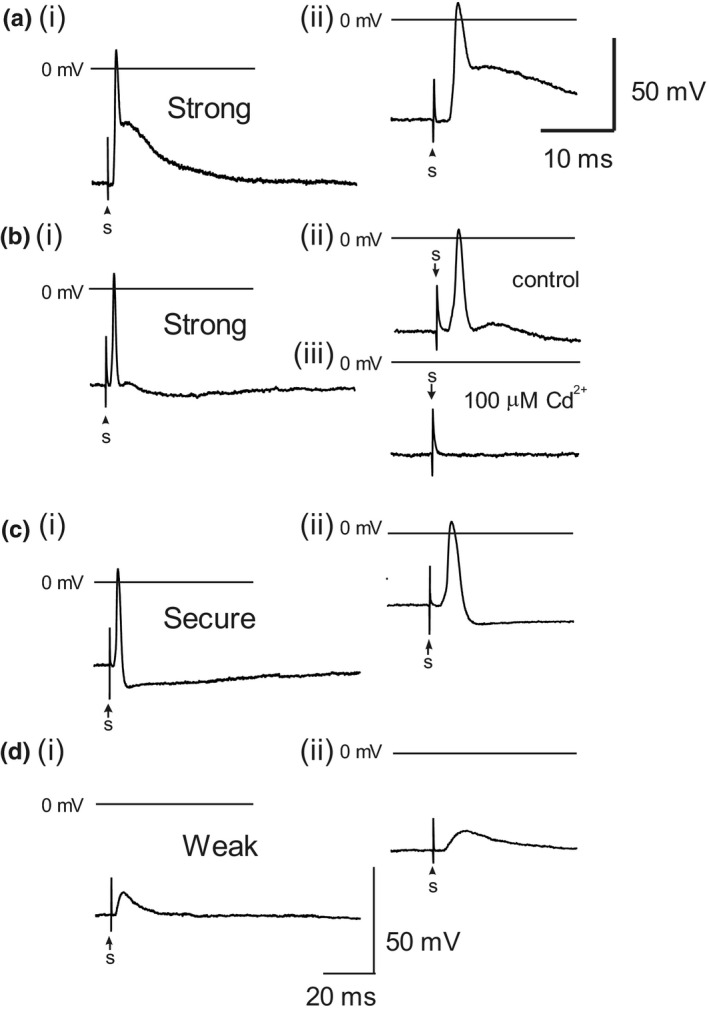
Representative neurally evoked postsynaptic responses illustrating the range of strength of synaptic transmission. Traces displayed at two different time scales (i) 20 ms, and (ii) and (iii) 10 ms, stimulus applied at time 's'. (a, b) Strong ganglionic transmission, where the AP arises early during the EPSP which is apparent as a shoulder or inflexion following the AP or AHP. (b) (ii) and (iii) Cd^2+^ (100 µM) blocks synaptic transmission. (c) Secure transmission where the AP arises late in the EPSP when present, or without an obvious EPSP. (d) Weak transmission

Ganglionic transmission was investigated for single stimuli at frequencies ≤0.5 Hz, low frequency stimuli at 0.5 Hz, and multiple trains of stimuli applied at 5–100 Hz. Synaptic responses to single stimuli were divided into three groups based on the synaptic response and AP waveform: *strong*, where the AP arises early during the excitatory postsynaptic potential (EPSP), *secure* where the AP arises late in the EPSP, when present, or without an obvious EPSP, and *weak* where nerve stimulation evokes a subthreshold EPSP. The scheme adopted for classification of synaptic strength was akin to that used for rat submandibular neurons (Smith et al., 1999). Representative examples of these synaptic responses are shown in Figure 5a–d. The three groups having the following relative frequency: strong 15/20, secure 4/20, and weak 1/20. In the case of the weak response, increasing stimulus intensity beyond the threshold required to recruit a synaptic potential did not change the amplitude of the evoked EPSP.

It has been reported that the vagus and interganglionic nerve connectives may contain postganglionic axons (Edwards et al., 1995). If so, APs evoked in these axons would be conducted antidromically to ICG neuron somata. To confirm that synaptic transmission was being investigated, the wide spectrum Ca^2+^ channel inhibitor, Cd^2+^ (100 µM), which blocks synaptic transmission (Smith et al., 1999), was routinely added to the superfusing PSS (see Figure 5b, ii and iii).

**FIGURE 6:**
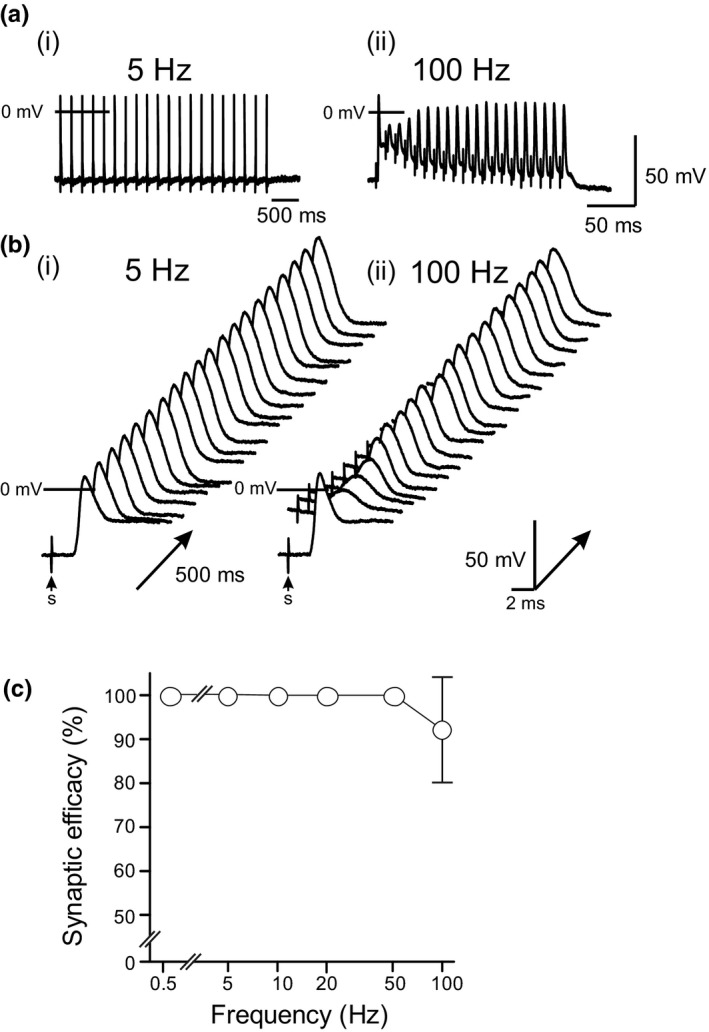
The ability of the postganglionic neuron to follow the activity of pre‐ganglionic stimuli at different frequencies (5–100 Hz). (a) Representative postsynaptic events evoked by trains of 20 stimuli at (i) 5 and (ii) 100 Hz. Note the different timescales for each applied frequency. (b) Waterfall displays of responses to 20 stimuli at (i) 5 and (ii) 100 Hz applied at time “s” are shown. (c) Synaptic efficacy was determined as the percentage of postsynaptic action potentials as a function of nerve stimulation frequency plotted on a logarithmic scale. Results are shown for 10 ICG neurons

The efficacy of synaptic transmission as a function of stimulation frequency was evaluated at 5, 10, 20, 50, and 100 Hz in 10 IGG neurons with strong postsynaptic responses. Trains of stimuli (20) were applied and AP discharge was monitored in the postganglionic neuronal soma. Synaptic efficacy was determined as the percentage of postsynaptic APs as a function of nerve stimulation. Recordings from a neuron in response to trains of 20 stimuli delivered to the vagus nerve trunk at 5 and 100 Hz are presented in Figure 6a,b. Ganglionic transmission was consistently faithful (100%) over the range 5–50 Hz, and decreased only minimally (92 ± 13%, *n* = 10) at 100 Hz stimulation (Figure 6c).

### 3.4 Conotoxin inhibition of synaptic transmission

**FIGURE 7:**
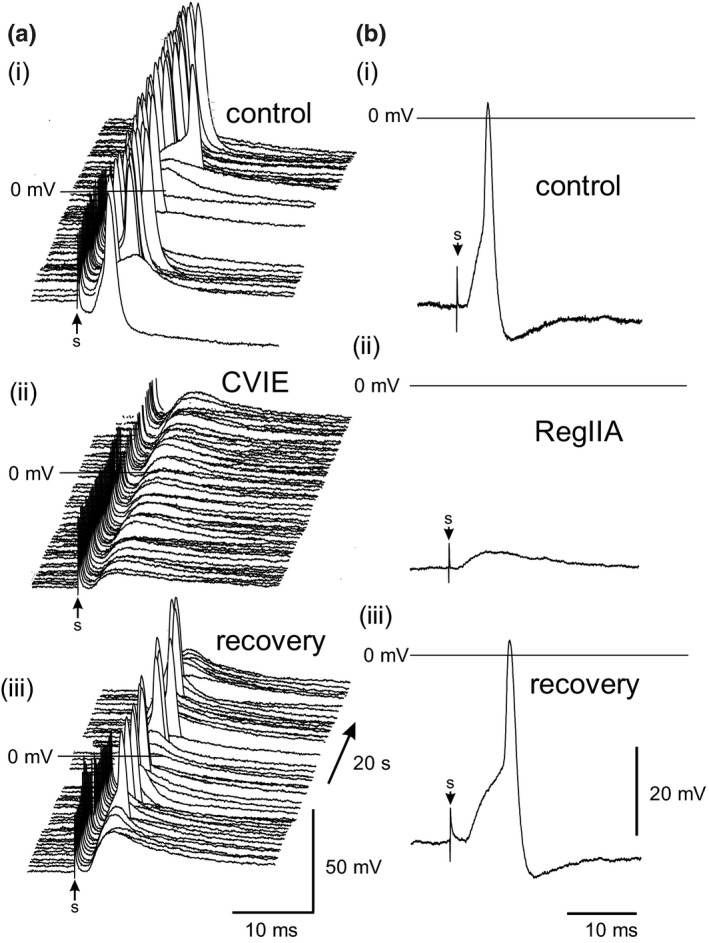
Inhibition of ganglionic transmission in the mouse ICG by selective antagonists of N‐type calcium (Cav2.2) channels (a) and neuronal nicotinic acetylcholine receptors (nAChRs) (b). (a) Waterfall displays of postsynaptic responses to 50 stimuli at 0.5 Hz applied at “s” are shown in the absence (control, washout) and presence of the selective N‐type (Cav2.2) calcium channel blocker CVIE (30 nM). (b) Representative traces of nerve‐evoked postsynaptic responses obtained in the absence (control) and presence of α‐conotoxin RegIIA (1 µM), a selective antagonist of α3‐containing nAChRs. Nerve stimulation 1 Hz, 0.02 ms duration applied at time “s” with postsynaptic inhibition by Reg IIA and recovery upon washout presented

Disulfide‐bonded peptides isolated from cone snail venom (conotoxins) have been used to investigate synaptic transmission in rat parasympathetic ganglia (Adams et al., 2003; Bibevski et al., 2000; Smith et al., 1999). The role of N‐type calcium (Cav2.2) channels in nerve‐evoked transmitter release in the mouse ICG was examined using ω‐conotoxin CVIE which has been shown to potently and selectively inhibit native and recombinant Cav2.2 channels (Berecki et al., 2010). Bath application of 30 nM CVIE reversibly inhibited synaptic transmission upon vagal nerve stimulation at 0.5 Hz (Figure 7a) consistent with presynaptic Cav2.2 channels in vagal nerve terminals having a dominant role in nerve‐evoked transmitter release.

α‐Conotoxins exhibit characteristic individual selectivity profiles for postsynaptic nicotinic acetylcholine receptors (nAChRs). The effect of a relatively potent and selective antagonist of α3‐containing nAChRs, α‐conotoxin RegIIA (Cuny et al., 2018; Franco et al., 2012), was examined on the nerve‐evoked postsynaptic response in mouse ICG neurons. α‐Conotoxin RegIIA (1 µM) was applied to three preparations with suprathreshold synaptic responses. In all ICG neurons studied, RegIIA blunted synaptic transmission by reversibly reducing the EPSP amplitude to approximately one‐third of control values but only completely blocked transmission in one preparation (Figure 7b). In the remaining neurons, RegIIA reversibly reduced the amplitude of the EPSP following the action potential to approximately ~0.3 of control values. Synaptic transmission and ACh‐evoked responses in rodent ICG have previously been shown to be completely and reversibly blocked by the ganglionic nAChR antagonists, mecamylamine and hexamethonium (Seabrook et al., 1990; Selyanko & Skok, 1992; Wang et al., 2002). α‐Conotoxin ImI, primarily selective for the α7 nAChR subtype, had no significant effect on ACh‐evoked currents in mouse ICG neurons when bath applied at 1 μM (*n* ≥ 4). This is consistent with the observation that mice deficient in the α7 subunit do not display differences in heart rate responses compared to control (Deck et al., 2005) indicating that the α7 subunit does not contribute critically to resting parasympathetic control of the heart.

## 4 DISCUSSION

In this study, we describe the passive and active membrane properties of adult mouse ICG neurons and synaptic transmission in mouse ICG using a whole‐mount preparation of the excised right atrial ganglion plexus. The experiments were performed at a temperature, divalent ion concentration, and pH buffering system relevant to *in vivo* conditions. The extrinsic cardiac nerves access the mouse heart at the right and left cranial veins and interblend within the ganglionated nerve plexus of the heart hilum that is localized on the heart base. The majority of intrinsic cardiac ganglia are localized on the heart base at the roots of the pulmonary veins. These ganglia are interlinked by interganglionic nerves into the nerve plexus of the heart hilum. Despite substantial anatomic differences in ganglion number and distribution, the structural organization of the intrinsic ganglionated plexus in the mouse heart corresponds in general to that of other mammalian species, including human (Rysevaite, Saburkina, Pauziene, Noujaim, et al., 2011; Rysevaite, Saburkina, Pauziene, Vaitkevicius, et al., 2011).

The cell capacitance of mouse ICG neurons was 27.7 ± 9.3 pF which is much less than that reported for adult rat ICG neurons, but similar to that for neurons in neonatal (postnatal 2–5 day) rats. In line with this, the mean input resistance (197.2 MΩ) and time constant (5.8 ms) values for adult mouse ICG neurons are akin to that for neonatal rat ICG neurons (Rimmer & Harper, 2006) which in all probability mirrors the size of the neurons.

Hyperpolarizing current pulses induced voltage‐ and time‐dependent inward rectification in mouse ICG neurons similar to that observed in rat ICG neurons (Hogg et al., 2001). Membrane hyperpolarization reduced *R*_in_ ~two‐fold consistent with an increase in membrane conductance most likely due to a hyperpolarization‐activated nonselective cation current, *I*_H_, and/or an inwardly rectifying K^+^ current, *I*_Kir_ (Cuevas et al., 1997; Hogg et al., 2001).

Adult murine ICG neurons exhibited predominantly phasic discharge in response to long depolarizing current pulses. It has been demonstrated for sympathetic ganglion neurons that the firing behavior evoked by depolarizing currents is correlated with the efferent activity requirements of the tissue innervated by the neuron (Cassell et al., 1986). Extending this to ICG neurons, the discharge properties of the somatic membrane reflect the requirements for a fast changing parasympathetic regulation of cardiac pacemaker activity.

Somatic APs evoked by brief depolarizing current pulses had an overshoot of >+15 mV and an AHP amplitude of 10 mV and half duration of ~45 ms. In a small population (<25%) of ICG neurons, spontaneous excitatory synaptic activity was observed and, less frequently, spontaneous action potentials. No pacemaker‐like action potential discharge activity was observed consistent with that reported for rat ICG neurons (Rimmer & Harper, 2006).

The analysis of ganglionic transmission in this study was limited to neurons displaying a consistent postsynaptic potential response to nerve (vagus) stimulation. There are at least two different neuron types within the ICG of adult mammals identified according to the nature of the synaptic input they receive. The neurons which receive a direct efferent projection from the vagus are classed principal neurons (McAllen et al., 2011) and those that receive a local excitatory synaptic input, secondary to a robust excitatory efferent projection from vagal preganglionic fibers, are categorized as interneurons. In addition, it is held that there are local or centrally projecting sensory neurons, which do not receive a synaptic input from the vagus or local connectives (Edwards et al., 1995). Intracellular recording from ICG postganglionic neurons of the WHBP of rats indicate that ~40% of the neurons are synaptically activated by preganglionic parasympathetic cardiomotor (principal) neurons, whereas the remaining 60% of ICG neurons cannot be activated by vagal preganglionic neurons and are silent (McAllen et al., 2011).

In this study, recordings were from what would be classed as principal ICG neurons, in receipt of a short delay excitatory input from the vagus. Neurons not activated by preganglionic inputs were disregarded. In the cohort of neurons examined, there were no instances of a delayed excitatory synaptic response consistent with that anticipated from interneurons, S cells according to the classification of Edwards et al. (1995). Thus, these neurons would be classed as SAH or principal cardiac neurons according to the electrophysiological and morphological schemes of Edwards et al. (1995), McAllen et al. (2011), and Cheng and Powley (2000), respectively.

Synaptic responses to single stimuli of the vagus or interganglionic nerves were divided into three groups: 75% *strong*, 20% *secure*, and 5% *weak*. The synaptic responses (EPSPs and APs) were blocked by extracellular Cd^2+^ consistent with synaptic transmission and not antidromic conduction. The efficacy of synaptic transmission as a function of stimulation frequency studied in neurons with strong synaptic responses was 100% at 5–50 Hz and decreased slightly to ~92% at 100 Hz stimulation. Given that the resting heart rate in the conscious unrestrained mouse is 500–700 beats per minute and that it can increase by 50% during strenuous exercise (Doevendans et al., 1998; Janssen et al., 2016), then synaptic transmission would be expected to be secure in the range of 5–50 Hz vagal stimulation. In this study, the number of repetitive pulses delivered in a train was 20 whereas in some reports this can be as much as 100 (Smith et al., 2016). This may impact on the synaptic efficacy of ganglionic transmission and requires further investigation of the muscarinic response particularly with regard overflow to extrajunctional receptors in cardiac muscle (Demir et al., 1999).

The presynaptic calcium channels and postsynaptic nAChRs involved in synaptic transmission in mouse ICG were identified using ω‐ and α‐conotoxins selective for voltage‐gated calcium channels and neuronal nAChRs, respectively. Vagus nerve‐evoked transmitter release was reversibly inhibited by ω‐conotoxin CVIE suggesting that presynaptic N‐type (Cav2.2) calcium channels largely contribute to transmitter release. Although multiple neuronal nAChR subtypes are expressed in ICG neurons (Bibevski et al., 2000; Poth et al., 1997), the >65% inhibition of the nerve‐evoked postsynaptic response by RegIIA suggests that the α3‐containing nAChR subtypes are the predominant neuronal nAChR functionally expressed in mouse ICG neurons. α‐Conotoxin RegIIA inhibits rat α3β2 and α3β4 nAChRs with an IC_50_ of 33 and 97 nM, respectively (Cuny et al., 2018). Interestingly, α‐conotoxin AuIB (1 μM), an antagonist selective for rat nAChRs containing an α3/β4 subunit interface (Luo et al., 1998), had been shown to attenuate sinus cycle length responses (∼20%) in canine intracardiac ganglion in situ (Bibevski et al., 2000). However, in adult rat atrial ganglionated plexus, AuIB (3 µM) had no effect on either synaptic transmission (0.2–50 Hz) or excitatory ACh‐evoked membrane potential responses (Adams et al., 2013).

Impaired parasympathetic control of the heart is a powerful, independent prognostic predictor of arrhythmia and also a characteristic of myocardial infarction and heart failure. Abnormalities in ganglionic transmission in a heart failure model (canines with heart failure induced by rapid ventricular pacing) have been identified as the site of this dysfunction (Arora et al., 2003; Bibevski & Dunlap, 1999, 2004). An indication that decreased synaptic transmission in ICG contributes to abnormal parasympathetic function in hypertension comes from experimental models (Heaton et al., 2007). Reduction in transmission in the ICG will produce a sympathovagal imbalance and thus a predominance of sympathetic, pro‐arrhythmic activity. The ICG has been documented as the site of attenuated vagal control of the heart and associated poor outcomes in patients following myocardial infarction and heart failure (Bibevski & Dunlap, 2011).

The results from this study will provide a framework for the use of transgenesis and/or gene targeting to delimit the genes whose functions determine integration of neuronal signaling within these ganglia. Empirically based mathematical models of the intracardiac neuronal plexus will be developed, allowing schemes for the mechanisms of ganglionic neuronal integration to be investigated.

## CONFLICT OF INTEREST

The authors declared that there is no conflict of interest, financial or otherwise.

## AUTHOR CONTRIBUTIONS

A.A.H. and D.J.A. conceived and designed research; A.A.H. performed experiments; A.A.H. and D.J.A. analyzed data and interpreted results of experiments; A.A.H. prepared figures; A.A.H. and D.J.A. drafted manuscript, edited, and revised manuscript; A.A.H. and D.J.A. approved final version of manuscript.

## Data Availability

The data that support the findings of this study are available from the corresponding author upon reasonable request.
